# Factors related to health-related quality of life in older people with multimorbidity and high health care consumption over a two-year period

**DOI:** 10.1186/s12877-019-1194-z

**Published:** 2019-07-05

**Authors:** Leonie Klompstra, Anne W. Ekdahl, Barbro Krevers, Anna Milberg, Jeanette Eckerblad

**Affiliations:** 1 Department of Social and Welfare Studies, Division of Nursing, Linköping University, Linköping, Sweden; 2 Section of Geriatric Medicine and Institution of Clinical Research, Helsingborg Hospital, Lund University, Lund, Sweden; 3 Department of Medicine and Health Sciences, Division of Health Care Analysis, Linköping University, Linkoping, Sweden; 4 Department of Advanced Home Care, Linköping University, Norrköping, Sweden; 5 Department of Medicine and Health Sciences, Linköping University, Linköping, Sweden; 6 Department on Neurobiology and Care Science and Society, Division of Nursing, Karolinska Institute, Stockholm, Sweden; 7 Department of Social and Welfare Studies, Linköping University, SE 601 74 Norrköping, Sweden

**Keywords:** Comprehensive geriatric assessment, Ambulatory geriatric care, Multimorbidity, High health care consumption, HrQoL, Symptom burden

## Abstract

**Background:**

The prevalence of multimorbidity is increasing worldwide, and older people with multimorbidity are frequent users of health care services. Since multimorbidity has a significant negative impact on Health-related Quality of Life (HrQoL) and is more common in older age it would be expected that factors related to HrQoL in this group might have been thoroughly researched, but this is not the case. Furthermore, it is important to look at old people living at home, considering the shift from residential to home-based care. Therefore, we aim to investigate factors that are related to HrQoL in older people with multimorbidity and high health care consumption, living at home.

**Methods:**

This is a secondary analysis of a RCT study conducted in a municipality in south-eastern Sweden. The study had a longitudinal design with a two-year follow-up period assessing HrQoL, symptom burden, activities of daily living, physical activity and depression.

**Results:**

In total, 238 older people with multimorbidity and high health care consumption, living at home were included (mean age 82, 52% female). A multiple linear regression model including symptom burden, activities of daily living and depression as independent variables explained 64% of the HrQoL. Higher symptom burden, lower ability in activities of daily living and a higher degree of depression were negatively related to HrQoL. Depression at baseline and a change in symptom burden over a two-year period explained 28% of the change in HrQoL over a two-year period variability. A higher degree of depression at baseline and negative change in higher symptom burden were related to a decrease in HrQoL over a two-year period.

**Conclusion:**

In order to facilitate better delivery of appropriate health care to older people with high health care consumption living at home it is important to assess HrQoL, and HrQoL over time. Symptom burden, activities of daily living, depression and change in symptom burden over time are important indicators for HrQoL.

**Trial registration:**

Clinicaltrials.gov identifier: NCT01446757, the trial was registered prospectively with the date of trial registration October 5^th^, 2011.

## Background

Multimorbidity is a state with co-occurrence of multiple chronic diseases in the same person and is common, with a prevalence estimate of 65–98% in older people [1–3]. The prevalence of multimorbidity is increasing worldwide due to the effects of better medical care and an ageing population [4]. A shift from the traditional single disease paradigm to a more holistic patient-centred approach [1, 2, 5, 6], including a focus on multiple diseases, is considered to be one of the greatest challenges for the society of this century [7].

Older people with multimorbidity are frequent users of health care services, and there is a relationship between the number of chronic conditions and health care costs [8, 9]. In addition, multimorbidity increases the risk of mortality and functional decline, and this negatively impacts health-related quality of life (HrQoL) [2, 10, 11]. Furthermore, research tends to focus on individual organ systems, often ignoring the complexity of care for older people with multimorbidity [12].

Older people with multimorbidity have been reported to suffer from a high symptom burden such as pain, dry mouth, lack of energy, and difficulty in sleeping, and such symptom burden has a negative effect on HrQL [13–16].Additionally, high symptom burden, pain in particular, often leads to a limitation of physical activity with a decrease in physical functioning that is negatively related to ability in the activities of daily living [17, 18].

Participation in physical activity promotes healthy aging in older people and plays an important role in improving HrQoL [19–21]. Older people who are physically active have lower rates of all-cause mortality and a higher level of muscular fitness compared with older people who are less physically active [19]. Therefore, increasing physical activity among older people has become an international priority [19, 21].

Although mental health is assumed to have a significant effect on HRQoL, it has been difficult to define its precise role. However, available evidence suggests that psychological variables such as depression have effects on many of the components of HRQoL [22, 23].

Since multimorbidity has a significant negative impact on HrQoL and is more common in older age, it would be expected that this relationship would have been thoroughly researched, but this is not the case, mainly due to a regular exclusion of older people with multimorbidity from ongoing trials [24, 25]. There is a shift from residential to home-based care as a potentially more effective and financially sustainable approach to meeting the health and social care needs of older people. Therefore, it is important to assess the needs of older people living at home. To our knowledge, no studies have shown which factors are associated with changes in HrQoL in older people with multimorbidity living at home and in order to prevent a decline in HrQOL it is important to identify what factors are important to assess and manage.

Therefore, we aim to investigate factors that are related to HrQoL in older people living at home with three or more diseases and high health care consumption, and factors that are related to change in HrQoL over a two-year period. We hypothesized that in older people with multimorbidity and high health care consumption, symptom burden, limitations of activities of daily living, limitations in physical activity and depression are possible factors related to HrQoL.

## Method

This is a secondary analysis using data from a RCT study (AGe-FIT), that was aimed at examining the costs and effects of care based on a comprehensive geriatric assessment provided by an ambulatory geriatric care unit in addition to usual care [26]. The Age-FIT study included a selected group of old people with multimorbidity, namely community-dwelling older people, 75 years or older, who had received in-patient hospital care three or more times in the previous year and had three or more diagnoses according to ICD-10. Data were collected between February 2011 and December 2013 in a municipality in south-eastern Sweden, which contains rural and urban areas and had approximately 130,000 inhabitants, 8.3% of whom were 75 years or older. In the original AGe-Fit study, 382 individuals were included and 252 completed the 2 year follow up. In this longitudinal study, we excluded older people who were not living at home after 2 years (*n* = 14), for this reason, the current analysis included data of 238 older people with multimorbidity, living at home.

Eligible older people received an invitation letter by post and were contacted by telephone to provide verbal informed consent to participate in the study. A registered nurse or an occupational therapist made home visits to obtain written informed consent and to collect data.

The present study complies with the Declaration of Helsinki and was approved by the Regional Ethics Committee in Linköping (Regionala Etikprövningsnämnden i Linköping), Sweden (Dnr. 2011/41–31). The trial was registered at clinicaltrials.gov, identifier NCT01446757.

### Measurements

Interviews were conducted to collect background and clinical data (e.g. age, sex, educational level, cohabitation, cognition) and to assess HrQoL, symptom burden, activities in daily living and depression at baseline and two-year follow-up.

*HrQoL* was measured by the Nottingham Health Profile Part I [27]. This instrument measures perceived health problems and the extent to which these problems affect normal daily activities. The questionnaire comprises 38 items and evaluates six dimensions of the health statuses of individuals: energy (three items), pain (eight items), emotional reactions (nine items), sleep (five items), social isolation (five items) and physical activity (eight items). The questions require yes/no answers. Each section is scored between 0 and 100, where 0 represents the best health status and 100 represent the worst health status. Change in HrQoL was the difference in the total score after 2 years compared to baseline. The Swedish version is found to be valid and reliable [28, 29].

*Symptom burden* was measured with the Memorial Symptom Assessment Scale [15, 30]. Theis instrument includes 24 symptoms that were evaluated with respect to frequency, severity, and distress. A further eight symptoms are evaluated in terms of severity and distress. Each symptom is recorded as being either present or absent and, if present, is rated using a four-point rating scale (1–4) for frequency and severity, and a five-point scale (0–4) recording distress during the previous 7 days, with higher scores indicating greater frequency, more severity, and higher distress. If a symptom is present, the symptom score is an average of the total of all scores within these dimensions. Change in symptom burden was a change in the total score after 2 years compared to baseline. The Swedish version has been found to be valid and reliable [31, 32].

*Activities in daily living* were measured by the Barthel Activities of Daily Living Index [33]. The index measures the current ability in each of the following activities: bathing, grooming, dressing, feeding, toilet use, urinary and faecal incontinence, transferring, walking 50 m and stair use. The items ranged from 0 (total disability in all activities of daily living) to 100 (ability/ in all activities of daily living). Change in activities in daily living was a change between the total score after 2 years compared to baseline. The index is valid and reliable [34].

*Physical activity* was assessed by the Short form International Physical Activity Questionnaire in Metabolic Equivalent of Task (Mets). This instrument contains seven questions for identifying the frequency and duration of light (< 600 Mets/week; walking at work and at home, walking from place to place, and any other walking that might be done solely for recreation, sport, exercise, or leisure), moderate (between 600 and 3000 Mets/week; carrying light loads, cycling at a regular pace, or doubles tennis), and vigorous (> 3000 Mets/week; lifting, digging, aerobics or fast cycling) physical activity as well as inactivity during the past week. The total physical activity score is the sum of vigorous, moderate, and walking in Mets in the past week. Change in physical activity was a change between the total score after 2 years compared to baseline. Typical correlations of this instrument with an accelerometer were 0.80 for reliability [35, 36].

*Depression* was assessed with the 15-item Geriatric Depression Scale [37, 38]. This scale contains 15 yes-no items, which were combined into a composite by counting the number of depressive responses. Scores ≥5 could indicate clinically important depressive symptoms [39]. Change in depression was a change between the total score after 2 years compared to baseline. The instrument is valid and reliable and also shown to be feasible for adults with cognitive dysfunction [39].

### Statistical analyses

Statistical analyses were performed using the Statistical Package for the Social Science version 25 (SPSS, Chicago, Illinois). In the descriptive analyses, means and standard deviations were calculated for continuous data, and absolute numbers and percentages were computed for nominal variables. Normal distribution of the data was tested by the Kolmogorov-Smirnov test. Data that was not normal distributed were presented with median and InterQuartile Range (IQR) and normal distributed data with mean and standard deviation.

### Statistical analysis for HrQoL and change in HrQoL over a two-year period

Because HrQoL was not normally distributed, the Wilcoxon Signed rank test was used to investigate differences between the HrQoL at baseline compared to 2 years.

Spearman’s correlations were conducted between HrQoL at baseline with the factors to consider as independent variables in the regression model (age, sex, cognition, symptom burden, physical activity, activities of daily living, and depression).

Spearman’s correlations were also conducted between difference in HrQoL at two-year compared to baseline with the factors to consider as independent variables in the regression model (age, sex, cognition, symptom burden, physical activity, activities of daily living, depression and change over a two-year period in symptom burden, physical activity, activities of daily living and depression).

Multiple regression models were built by entering those variables that had were statistical significance with a *p* < 0.05 in the correlation and retaining variables in the final regression with a significant level of *p* < 0.05.

## Results

In total, 238 participants with multimorbidity and high health care consumption, living at home, were included in this study. The mean age was 82 (75–96) years and more than half were female (52%). The participants in this study had a variety of different diagnoses in their medical record, and almost all (94%) had diseases of the circulatory system, 79% had diseases of the musculoskeletal system and more than 50% had diseases within the eye and adnexa, the digestive system and the respiratory system. The participants could also have different diagnosis within each ICD − 10 chapter (Table 1) cognition was mean 27.80 (±2.74). Of the participants, 91% had a normal cognitive function (*n* = 195).

**Table 1:** Sociodemographic of 238 older people with multi-morbidity and high health care consumption, living at home

Age (years) mean	82 ± 4
Female gender	123 (52%)
Living alone	119 (50%)
Educational level	
-Primary school	142 (69%)
-Secondary school	40 (17%)
-Higher education/University	36 (15%)
Normal cognitive function	195 (91%)
Diagnosis according to ICD 10	
01. Certain infectious and parasitic diseases (A00-B99)	107 (45%)
02. Neoplasma (C00-D48)	95 (40%)
03. Diseases of the blood and blood-forming organs and certain disorders Involving the immune mechanism (D50-D89)	62 (26%)
04. Endocrine, nutritional and metabolic diseases (E00-E90)	114 (48%)
05. Mental and behavioral disorders (F00-F99)	73 (31%)
06. Diseases of the nervous system (G00-G99)	81 (34%)
07. Diseases of the eye and adnexa (H00-H59)	140 (59%)
08. Diseases of the ear and mastoid process (H60-H95)	84 (35%)
09. Diseases of the circulatory system (I00-I99)	225 (94%)
10. Diseases of the respiratory system (J00-J99)	120 (50%)
11. Diseases of the digestive system (K00-K93)	126 (53%)
12. Diseases of the skin and subcutaneous tissue (L00-L99)	99 (42%)
13. Diseases of the musculoskeletal system and connective tissue (M00- M99)	187 (79%)

*ICD* International classification of diseases, *SD* Standard deviation

At baseline, the participants scored a HrQoL of a median of 20 (IQR 8–33) and on the subscales of HrQoL; energy level median 24 (IQR 0–63), pain median 10 (IQR 0–34), emotional reaction median 7 (IQR 0–23), sleep median 22 (0–38), social isolation median 0 (IQR 0–22) and physical abilities median 22 (IQR 11–42). The symptom burden was at baseline mean 0.61 (±.41), activities of daily living was mean 92 (±11) the physical activity was 724.50 Mets (±2463.14), depression was mean 3.47 (±3.01).

### Health-related quality of life

Lower HrQoL was significantly related to higher age (*r* = .062), higher symptom burden (*r* = .719) and higher depression (*r* = .651) (*P* < .001). Being male (*r* = −.224), engaging in higher physical activity (*r* = −.374) and showing higher ability in activities of daily living (*r* = −.437) were related to a better HrQoL (*P* < .001) (Table 2).

**Table 2:** Correlation matrix of Health related Quality of Life with age, gender, cognition, symptom burden, physical activity, activities of daily living and depression

	HrQoL	Age	Gender	Cognition	Symptom burden	Physical activity	Activities of daily living	Depression
HrQoL	1							
Age	.062**	1						
Gender^a^	−.224**	−.106	1					
Cognition	−.113	−.163	−.106	1				
Symptom burden	.719**	.063	.231**	−.064	1			
Physical activity	−.374**	−.015	.209**	.143*	−.196**	1		
Activities of daily living	−.437**	−.034	.204**	.124	.275**	.436**	1	
Depression	.651**	.050	−.165*	−.139*	.553**	−.294*	−.367**	1

*HrQoL* Health related quality of life

* *p* < .005 ** *p* < .001

^a^ variable dichotomized: 0 = female; 1 = male

Age, gender, cognition and physical activity were not factors related to HrQoL in the regression analysis. Taking these variables out of the regression analyses, a higher symptom burden, lower ability in activities of daily living and more depressive symptoms explained 64% of the variability in negative changes in HrQoL (Table 3).

**Table 3:** Factors (symptom burden, activities of daily living and depression) related to Health related Quality of Life. Age, gender, cognition and physical activity were not factors related to HrQoL in the regression analysis

Variables	Unstandardized *ß*	Standardized *ß*	*P*-value	VIF	95% Confidence interval		*R* ^2^	*F*
Symptom burden	17.39	0.44	< 0.01	1.59	12.96	21.83		
Activities of daily living	−0.40	−0.20	< 0.01	1.20	−0.59	−0.21		
Depression	2.06	−0.35	< 0.01	1.71	1.38	2.74		
							0.64	107.73

### Changes over a 2-year period in health-related quality of life

The HrQoL of the participants changed positively over a two-year period. At baseline, the participants scored a median of 20 (IQR 8–33) and after 2 years 15 (IQR 6–30) (*P* = 0.04). Looking at individual dimensions of HrQoL, one change was found: the quality of sleep was better after 2 years with a median of 13 (IQR 0–35) compared to baseline with a median of 22 (IQR 0–38 (*P* = 0.02).

The following variables were correlated with a negative change in HrQoL over a 2-year period: negative change in symptom burden (experiencing higher symptoms burden) at 2 years compared to baseline (−.471), lower abilities in activities of daily living at baseline (*r* = −.187), higher depression at baseline (*r* = .236) and more depressive symptoms at 2 years compared to baseline (−.241) (Table 4).

**Table 4:** Correlation matrix of change in Health related Quality of Life in a 2-year period with age, gender, cognition, symptom burden, physical activity, activities of daily living and depression and change over a 2-year period in symptom burden, physical activity, independence of daily living and depression

	Change in HrQoL	Age	Gender	Cognition	Symptom burden	Physical activity	Activities of daily living	Depression	Change in symptom burden	Change in physical activity	Change in activity of daily living	Change in depression
Change in HrQoL	1											
Age	.009	1										
Gender^a^	−.100	−.106	1									
Cognition	−.040	−.163*	−.106	1								
Symptom burden	.271**	.063	−.231**	−.064	1							
Physical activity	−.126	−.015	.209**	.143*	−.196**	1						
Activities of daily living	−.187*	−.034	.204**	.124	−.275**	.447**	1					
Depression	.236**	.050	−.165*	−.139*	.553*	−.369**	−.367**	1				
Change in symptom burden	−.471**	−.015	.032	−.148*	−.308**	.131*	−.060	−.161*	1			
Change in physical activity	−.000	−.018	−.001	.003	−.045	−.061	.202**	−.047	.078	1		
Change in activities of daily living	.139	−.021	.100	−.207*	−.055	−.022	−.215**	−.079	.237**	.047	1	
Change in depression	−.241**	−.061	.052	.017	−.205**	−.055	.049	−.271	−.149*	.062	.051	1

*HrQoL* Health related quality of life

* *p* < .005 ** *p* < .001

^a^ variable dichotomized: 0 = female; 1 = male

Age, gender, cognition, symptom burden at baseline, activities of daily living at baseline, and change in depression over two-year period were not factors related to change in HrQoL over a two-year period in the regression analysis and we took these out of the regression analysis. A negative change in symptom burden (experiencing higher symptoms burden) at 2 years compared to baseline, and higher experience of depressive symptoms at baseline, explained 28% of a decrease in HrQoL at 2 years compared to baseline (Table 5).

**Table 5:** Factors (depression and change in symptom burden) related to change in Health related Quality of Life over a 2 year period. Age, gender, cognition, symptom burden at baseline, activities of daily living at baseline, and change in depression were not factors related to change in HrQoL in the regression analysis

Variables	Unstanderdized *ß*	Standardized *ß*	*P*-value	VIF	95% Confidence interval		*R* ^2^	*F*
Depression at baseline	1.38	0.29	< 0.01	1.03	0.77	2.00		
Change in symptom burden	16.22	0.40	< 0.01	1.03	10.88	21.56		
							0.28	33.07

## Discussion

To our knowledge, this is the first study that assesses factors related to change over a two-year period of HrQoL in older people with multimorbidity living at home. Older people with multimorbidity experienced a better HrQoL over a two-year period, where quality of sleep contributed the most to a better HrQoL.

This study show that a higher symptom burden was a factor related to a lower HrQoL and a negative change in symptom burden (experiencing higher symptoms burden) at two-years compared to baseline was found to be a factor related to a decrease in HrQoL over a two-year period. A recent study including adults diagnosed with heart failure showed that, measured over a 12-month period, a reduced symptom burden was associated with a higher HrQOL [40]. Previous studies have shown the negative impact of symptom burden on HRQL in various chronic conditions such as renal failure [41], heart failure [42], COPD [43] and cancer [44]. Symptom burden has, however, mostly been studied from the perspective of a single disease, and more studies from the perspective of adults living with multimorbidity are needed.

Lower ability in activities of activities in daily living was found to be a factor related to a lower HrQoL in older people with multimorbidity. These results are supported by a previous study, where the impact of chronic disease on daily activities was found to be the most significant factor related to HRQoL outcomes [45], highlighting the importance of supporting older people with multimorbidity at home to maintain their abilities in activities of daily living.

Physical activity was not found as a factor related to HrQoL in the regressions or change in HrQoL over time in this study. A literature review [46] looking at the association between physical activity with HrQoL in elderly people found that physical activity may have an impact on some domains of HrQoL (functional capacity, general QoL; autonomy; past, present and future activities; death and dying; intimacy; mental health; vitality; and psychological). However, the association between physical activity and HrQoL and other HrQoL domains were moderate to inconsistent and require further investigation.

A higher degree of depression at baseline was a factor related to a lower HrQoL and a negative change in HrQoL over a two-year period. These findings are in line with the findings of the World Health Organization, where depression is seen as one of the most serious mental health disorders in older people [7]. Previous study revealed that the depressed older people had a lower HrQOL compared to those with many other chronic medical disorders [47].

According to our results, in order to identify persons with risk of low HrQoL we suggest that activities of daily living, symptom burden and depression should be assessed by health professionals taking care of older people with multimorbidity. Noticeably, the World Health Organization campaigned to focus on depression as a highlighted topic for the 2017 World Health Day [48]. Understanding factors that are related to HRQoL in older people with multimorbidity can be used to inform health service planning, to accommodate patient needs and to reduce the burden of disease on older people and the health system.

### Limitations

One limitation of this study is the assessment of physical activity. Physical activity is measured using a self-reported questionnaire and, although used in an elderly population, the instrument is validated for adults between 18 and 69 years of age. Future studies should consider measuring physical activity objectively by using activity monitors. Furthermore, we looked at physical activity as a factor related to the total score of HrQoL, while from the literature it is known that physical activity may only be associated with certain domains of HrQoL.

This study population was a selected sample, including older adults with multimorbidity, living at home and with high health care consumption and can therefore not be generalized to all older adults with multimorbidity.

## Conclusion

Lower HrQoL was negatively associated by higher symptom burden, more depressive symptoms and lower activities of daily living in older people with multimorbidity and high health care consumption who were living at home. Furthermore, over a two-year period, a decrease in HrQoL was associated with experiencing a higher degree of depression at baseline and a higher symptom burden at two-years compared to baseline. This means, in order to facilitate better delivery of appropriate health care to this group, it could be helpful for health care professionals to assess depression and change in symptom burden over time.

## Data Availability

The datasets used and/or analysed during the current study are available from the corresponding author on reasonable request.

## Abbreviations

HrQoL: Health related Quality of Life
IQR: InterQuartile Range
Mets: Metabolic Equivalent of Task

## References

[1] Fortin M, Stewart M, Poitras M-E, Almirall J, Maddocks H (2012). A systematic review of prevalence studies on multimorbidity: toward a more uniform methodology. Ann Fam Med.

[2] Glynn LG, Valderas JM, Healy P (2011). The prevalence of multimorbidity in primary care and its effect on health care utilization and cost. Fam Pract.

[3] Yarnall AJ, Sayer AA, Clegg A, Rockwood K, Parker S, Hindle JV (2017). New horizons in multimorbidity in older adults. Age Ageing.

[4] Goodman RA, Posner SF, Huang ES, Parekh AK, Koh HK (2013). Peer reviewed: defining and measuring chronic conditions: imperatives for research, policy, program, and practice. Prev Chronic Dis.

[5] van Oostrom SH, Picavet HSJ, van Gelder BM (2012). Multimorbidity and comorbidity in the Dutch population–data from general practices. BMC Public Health.

[6] Smith SM, Soubhi H, Fortin M, Hudon C, O’Dowd T (2012). Managing patients with multimorbidity: systematic review of interventions in primary care and community settings. BMJ.

[7] WHO (2014). Global status report on noncommunicable diseases 2014.

[8] Wolff JL, Starfield B, Anderson G (2002). Prevalence, expenditures, and complications of multiple chronic conditions in the elderly. Arch Intern Med.

[9] Lehnert T, Heider D, Leicht H (2011). Health care utilization and costs of elderly persons with multiple chronic conditions. Med Care Res Rev.

[10] Vogeli C, Shields AE, Lee TA (2007). Multiple chronic conditions: prevalence, health consequences, and implications for quality, care management, and costs. J Gen Intern Med.

[11] Marengoni A, Angleman S, Melis R (2011). Aging with multimorbidity: a systematic review of the literature. Ageing Res Rev.

[12] Applegate WB, Ip E (2016). The evolving taxonomy of health in older persons. JAMA.

[13] Pisu M, Azuero A, Halilova KI (2018). Most impactful factors on the health-related quality of life of a geriatric population with cancer. Cancer.

[14] Walke LM, Byers AL, Gallo WT, Endrass J, Fried TR (2007). The association of symptoms with health outcomes in chronically ill adults. J Pain Symptom Manag.

[15] Zambroski CH, Moser DK, Bhat G, Ziegler C (2005). Impact of symptom prevalence and symtom burden on quality of life in patients with heart failure. Eur J Cardiovasc Nurs.

[16] Eckerblad J, Theander K, Ekdahl A (2015). Symptom burden in community-dwelling older people with multimorbidity: a cross-sectional study. BMC Geriatr.

[17] Vaughan L, Leng X, La Monte MJ, Tindle HA, Cochrane BB, Shumaker SA (2016). Functional Independence in late-life: maintaining physical functioning in older adulthood predicts daily life function after age 80. J Gerontol A.

[18] Molton IR, Terrill AL (2014). Overview of persistent pain in older adults. Am Psychol.

[19] World Health Organisation. Global recommendations on physical activity for health. Geneva: World Health Organisation; 2010. 26180873

[20] Chodzko-Zajko WJ, Proctor DN, Singh MAF (2009). Exercise and physical activity for older adults. Med Sci Sports Exerc.

[21] Paterson DH, Jones GR, Rice CL (2007). Ageing and physical activity: evidence to develop exercise recommendations for older adults. Appl Physiol Nutr Metab.

[22] Lin J-H, Huang M-W, Wang D-W (2014). Late-life depression and quality of life in a geriatric evaluation and management unit: an exploratory study. BMC Geriatr.

[23] Chang-Quan H, Xue-Mei Z, Bi-Rong D, Zhen-Chan L, Ji-Rong Y, Qing-Xiu L (2009). Health status and risk for depression among the elderly: a meta-analysis of published literature. Age Ageing.

[24] Cherubini A, Oristrell J, Pla X (2011). The persistent exclusion of older patients from ongoing clinical trials regarding heart failure. Arch Intern Med.

[25] Zulman DM, Sussman JB, Chen X, Cigolle CT, Blaum CS, Hayward RA (2011). Examining the evidence: a systematic review of the inclusion and analysis of older adults in randomized controlled trials. J Gen Intern Med.

[26] Ekdahl AW, Wirehn AB, Alwin J (2015). Costs and effects of an ambulatory geriatric unit (the AGe-FIT study): a randomized controlled trial. J Am Med Dir Assoc.

[27] Hunt SM, McEwen J (1980). The development of a subjective health indicator. Sociol Health Illn.

[28] Wiklund I, Romanus B, Hunt SM (1988). Self-assessed disability in patients with arthrosis of the hip joint. Reliability of the Swedish version of the Nottingham health profile. Int Disabil Stud.

[29] Carlsson E, Olsson SB, Hertervig E (2002). The role of the nurse in enhancing quality of life in patients with an implantable cardioverter-defibrillator: the Swedish experience. Prog Cardiovasc Nurs.

[30] Portenoy RK, Thaler HT, Kornblith AB (1994). The memorial symptom assessment scale: an instrument for the evaluation of symptom prevalence, characteristics and distress. Eur J Cancer.

[31] Browall M, Kenne Sarenmalm E, Nasic S, Wengstrom Y, Gaston-Johansson F (2013). Validity and reliability of the Swedish version of the memorial symptom assessment scale (MSAS): an instrument for the evaluation of symptom prevalence, characteristics, and distress. J Pain Symptom Manag.

[32] Pettersson G, Berterö C, Unosson M, Börjeson S (2014). Symptom prevalence, frequency, severity, and distress during chemotherapy for patients with colorectal cancer. Support Care Cancer.

[33] Mahoney FI, Barthel DW (1965). Functional evaluation: the Barthel index. Md State Med J.

[34] Sulter G, Steen C, De Keyser J (1999). Use of the Barthel index and modified Rankin scale in acute stroke trials. Stroke.

[35] Hurtig-Wennlöf A, Hagströmer M, Olsson LA (2010). The international physical activity questionnaire modified for the elderly: aspects of validity and feasibility. Public Health Nutr.

[36] Craig CL, Marshall AL, Sjostrom M (2003). International physical activity questionnaire: 12-country reliability and validity. Med Sci Sports Exerc.

[37] Yesavage JA, Brink TL, Rose TL (1982). Development and validation of a geriatric depression screening scale: a preliminary report. J Psychiatr Res.

[38] D'Ath P, Katona P, Mullan E, Evans S, Katona C (1994). Screening, detection and management of depression in elderly primary care attenders. I: the acceptability and performance of the 15 item geriatric depression scale (GDS15) and the development of short versions. Fam Pract.

[39] Conradsson M, Rosendahl E, Littbrand H, Gustafson Y, Olofsson B, Lovheim H (2013). Usefulness of the geriatric depression scale 15-item version among very old people with and without cognitive impairment. Aging Ment Health.

[40] Heo Seongkum, Moser Debra K., Lennie Terry A., Fischer Mary, Kim JinShil, Lee Mikyoung, Walsh Mary N., Ounpraseuth Songthip (2018). Changes in Heart Failure Symptoms are Associated With Changes in Health-related Quality of Life Over 12 Months in Patients With Heart Failure. The Journal of Cardiovascular Nursing.

[41] Davison SN, Jhangri GS (2010). Impact of pain and symptom burden on the health-related quality of life of hemodialysis patients. J Pain Symptom Manag.

[42] Blinderman CD, Homel P, Billings JA, Portenoy RK, Tennstedt SL (2008). Symptom distress and quality of life in patients with advanced congestive heart failure. J Pain Symptom Manag.

[43] Blinderman CD, Homel P, Billings JA, Tennstedt S, Portenoy RK (2009). Symptom distress and quality of life in patients with advanced chronic obstructive pulmonary disease. J Pain Symptom Manag.

[44] Kenne Sarenmalm E, Browall M, Gaston-Johansson F (2014). Symptom burden clusters: a challenge for targeted symptom management. A longitudinal study examining symptom burden clusters in breast Cancer. J Pain Symptom Manag.

[45] Tyack Z, K-a F, Barnett A, Cornwell P, Kuys S, McPhail S (2016). Predictors of health-related quality of life in people with a complex chronic disease including multimorbidity: a longitudinal cohort study. Qual Life Res.

[46] Vagetti GC, Barbosa Filho VC, Moreira NB, Oliveira V, Mazzardo O, Campos W (2014). Association between physical activity and quality of life in the elderly: a systematic review, 2000-2012. Rev Bras Psiquiatr.

[47] Unutzer J (2009). Top cited papers in international psychogeriatrics: 2. Quality adjusted life years in older adults with depressive symptoms and chronic medical disorders. Int Psychogeriatr.

[48] (WHO) WHO (2016). Mental health and older adults.

